# The influence of positive affect on sensitivity to important omissions

**DOI:** 10.3389/fpsyg.2022.992489

**Published:** 2022-11-08

**Authors:** Susan P. Mantel, Tamara Montag-Smit, Frank R. Kardes, Alberto Barchetti

**Affiliations:** ^1^Marketing Department, Carl H. Lindner College of Business, University of Cincinnati, Cincinnati, OH, United States; ^2^Management Department, Manning School of Business, University of Massachusetts Lowell, Lowell, MA, United States

**Keywords:** omission neglect, positive affect, missing information, information processing, product evaluation

## Abstract

It is surprisingly difficult to notice when important information is missing (omission neglect) and yet, social media, advertisements, and other forms of communication typically only include one-sided information or positive attributes and omit opposing views or negative attributes. Even though it is surprisingly difficult to overcome this natural tendency, there are circumstances when decision makers are more sensitive to omissions. Understanding how and when decision makers can overcome this omission neglect tendency can be helpful to improve decision making in many situations. This paper investigates positive affect as a potential factor that can elicit sensitivity to omissions and alert decision makers to the need for additional information when important information is, in fact, missing. Four experiments use a consumer product choice situation to show that when decision makers are making an important decision, positive affect increases the likelihood that they will report a greater desire for additional product information (experiments 3 and 4) and temper their purchase interest in the target product. These results are shown using inference (experiments 1, 2, and 3) and by explicitly comparing a product choice with full and partial information (experiment 4). The results are discussed in terms of omission neglect literature as well as implications of the results for understanding the role of positive affect in information processing, judgment, and decision-making. These findings have implications for policy makers, marketers and others who are interested in message processing.

## Introduction

Every day, people are confronted with messaging on various platforms from social media to product advertisements that are designed to induce an action (e.g., purchase a product) or mold an opinion. Because of this, many decisions are made from messages that are designed to persuade by including only incomplete information and excluding important attributes or elements that are needed for a full analysis. Broad evidence shows that humans tend to go by what is in front of them, ignoring information that is not presented but potentially available (WYSIATI, or What You See Is All There Is; Kahneman, 2011). Cuing the decision maker has been shown to be effective in debiasing this type of association-based errors (Arkes, 1991) but this debiasing technique takes an overt action in order to alert the decision maker to the potential important missing information. For this reason, the study of how situational or personal characteristics can overcome omission neglect (or WYSIATI) can be important to public policymakers, marketers, and others.

According to the consumer behavior research on set-size effect (Anderson, 1981), when limited information is presented consumers should make moderate judgments with little confidence, because having less relevant information (i.e., fewer important product attributes presented) should decrease consumer confidence. Ideally, this would also result in a desire for additional product information in order to make a better decision. However, studies show that when consumers judge products singularly, they are not always sensitive to the number of important attributes presented (e.g., Kardes and Sanbonmatsu, 1993; Sanbonmatsu et al., 1997), meaning people form extreme judgments with high confidence even when important product information is missing. This phenomenon is known as omission neglect – or insensitivity to missing or unknown product options or features (Sanbonmatsu et al., 1991). Omission neglect occurs when people process a stimulus as if they had full information even when some important information is missing (inattention to missing information) or they make global inferences that all missing information is consistent with the presented attributes, attitudes, exemplars from the category, or activated schemata (Kardes et al., 2022).

According to the consumer information processing literature, when consumers begin to evaluate a stimulus, they will attend to, comprehend, and organize the available product information (including externally presented information as well as, in some cases, information stored in memory) into a cognitive representation (Kardes and Wyer, 2013). Omission neglect occurs when important, but missing, product information is not noticed (and therefore not included in information processing) because it is not readily available. That is, humans tend to go by what is in front of them (WYSIATI) and ignoring important information that is not available at the time of processing (Kahneman, 2011). This occurs because people, by default, are more likely to attend to the gestalt of the information presented rather than the specific features (Navon, 1977; Kahneman, 2011), and thus may be less able to recognize the absence of those specific features (e.g., Treisman and Souther, 1985).

Even so, to make a judgment, people must feel confident that they have the necessary (i.e., most important) information. Thus, when limited information is presented and important information is omitted, people may make superficial inferences about the missing information by assuming a correlation with the presented attributes (e.g., a beautiful product is assumed to perform better than a less beautiful product). These inferences, then, cause people to overestimate the importance of the presented information and underestimate the importance of potentially missing information, which results in increased certainty and extreme judgments (Sanbonmatsu et al., 2003). Thus, omission neglect occurs when people form strong beliefs on the basis of weak evidence (Sanbonmatsu et al., 1991, 1992, 1997, 2003; Caputo and Dunning, 2005).

Although omission neglect is commonly observed, omission detection (also referred to as sensitivity to omissions) can occur when consumers recognize that important information is missing from the product description, and they do not feel confident enough to make inferences about that information based on the information available to them. Hence, omission neglect is not inevitable, detection can occur in certain circumstances (Sanbonmatsu et al., 1991). For example, expertise with the product category increases the likelihood that a person will be able to detect missing product information (Sanbonmatsu et al., 1991, 1992). The category expert has a high familiarity with the product and therefore, is more likely to notice missing important information. Moreover, when people are highly engaged with a product category (e.g., bicycles), they are more likely to have well established criteria for evaluating products in the category and are better able to access memories about important information (e.g., comparable products) that should be considered. Thus, because they have a preconceived notion about the specific criteria that they need in order to make a judgment, they will feel greater uncertainty and be less influenced by the information presented if important attributes are missing (Kardes et al., 2006). This means they are also less likely to make superficial correlational inferences about the missing information and, instead, have a desire for additional information. Thus, the highly engaged person tends to evaluate products more moderately in the face of missing information (sensitivity to missing information).

When a product is generally familiar to most consumers, situational cues may influence their ability to detect missing information. For example, cognitive load (Sanbonmatsu et al., 1994), attribute alignability (Sanbonmatsu et al., 1997, 2003), and the salience of the missing information or the criteria to be applied to judgment (Kardes et al., 2006) have all been shown to influence the likelihood of omission detection. Adding to this body of research, we examine affective state as a new situational predictor of omission detection. Affect is particularly interesting because it has powerful effects on information processing and decision making (Isen, 2008; Cohen et al., 2018), and its influences are complex, nuanced, and malleable (Huntsinger, 2012; Huntsinger et al., 2014). Moreover, consumer affect is notably susceptible and influenced by even minor situational stimuli such as the context of ad placement, in-store promotions, or other stimuli in the environment (e.g., Machleit et al., 2000; Machleit and Mantel, 2001), which has resulted in a host of techniques used in stores to manipulate consumers’ feeling states (Gardner, 1985; Eroglu et al., 2003), including the use of pleasant music, warm ambient lighting, and free gifts to name a few.

## Positive affect and information processing

According to decades of research, one’s affective state has the power to influence the information processing style relied upon in a given situation, however, the specific style elicited has been debated (Pfister and Böhm, 2008). For example, one large body of research suggests that positive affect (compared to neutral or negative) facilitates heuristic information processing (e.g., Isbell et al., 2005), such that positive affect (compared to neutral) leads people to rely on general prior knowledge (e.g., Bless et al., 1996; Isbell, 2004; Bless and Burger, 2017) or categorical information (see Schwarz, 2011 for review, e.g., Bless et al., 1990) such as brand names (Adaval, 2003) and stereotypes (Bodenhausen, 1993; Bodenhausen et al., 1994), in addition to scripts and schemas (Bless et al., 1996) during judgment tasks. Generally, this research could suggest that positive affect increases one’s reliance on heuristic processing compared to negative and neutral affect.

The application of this stream of research to the omission neglect question is not clear cut. Some research suggests that if positive affect facilitates heuristic processing, individuals in a positive affective state should be more likely to use their mood as a heuristic cue and rate a product more favorably (i.e., mood congruence; Bodenhausen, 1993; Gorn et al., 1993; Martin et al., 1993; Bodenhausen et al., 1994; Pham, 1998). However, some evidence suggests that those in positive affect will focus some attention on the stored scripts and schemas resulting in a greater number of intrusion errors during memory recall, because they are more likely to use information from schematic scripts encoded in memory (Bless et al., 1996). In an omission neglect context, if positive affect participants are using heuristic processing style, we would expect them to exhibit omission neglect. However, if those in positive affect focus some attention on stored scripts and schemas, they might be alerted to potential important elements that are available in their stored schema, but not present in the attributes presented. This would suggest that those in positive affect would respond in a way that is similar to the response of category experts (Sanbonmatsu et al., 1991, 1992) and exhibit omission sensitivity.

Another large body of affect literature supports this later hypothesis. That is, positive affect has been shown to lead to detailed or analytical processing, especially when generating novel connections among disparate details (Ashby et al., 1999). In these studies, the positive affect participants were shown to be more likely to find the correct solution for a complex problem (Isen et al., 1987, e.g., Duncker candle task, Duncker and Lees, 1945), find the correct answer more quickly when switching between tasks (Wang et al., 2017), solve a clinical case study (Estrada et al., 1997), and focus on local, specific details (Isen et al., 1985). For example, when a list of specific words is presented, positive affect has been shown to enable people to generate more unusual and diverse first associates to neutral words (Isen et al., 1985; Kahn and Isen, 1993), to categorize objects more flexibly by grouping them into more diverse sets (Isen and Daubman, 1984; Murray et al., 1990), and to be better able to solve a wide range of problems (e.g., Carnevale and Isen, 1986; Estrada et al., 1997; Aspinwall, 1998; Erez and Isen, 2002). Positive moods also showed significant positive correlations with a growth mindset (Intasao and Hao, 2018), creativity (Han et al., 2019) as well as increased risk-taking (but not heightened impulsivity) tendencies (Herman et al., 2018). In fact, when performing a gambling task, those in a positive affect have been shown to have an increased tendency to take a gamble (Stanton et al., 2014) and performed better (i.e., an enhanced tendency toward choosing cards from the advantageous decks) on the Iowa Gambling Task (Vries et al., 2008). In a clinical situation, physicians in positive affect looked past simplistic inferences (e.g., less dependent on anchoring) when diagnosing a patient (Estrada et al., 1997). The result is more thoughtful cognitive inferences considering multiple possibilities. This is the explanation provided for the finding that positive affect participants are more likely to find the non-intuitively obvious solution to complex problems like the Duncker candle problem (Isen et al., 1987). It also leads to the expectation that when participants are in positive affect, they will focus on the concrete elements of the product information, leading to omission sensitivity, lower confidence, and moderate judgments.

In trying to decide between these two competing hypotheses (e.g., positive affect will cause omission neglect due to heuristic processing or positive affect will cause omission sensitivity due to detailed process), we can look to research that investigates when each type of processing occurs. For example, Krauth-Gruber and Ric (2000) found that when happy people are provided specific information that contradicts a stereotype, they do not rely on that stereotype to form judgments. In this case, they systematically process the detailed information provided, because the presentation of detailed information cues them to do so. However, the opposite effect is established when the information provided is not relevant to the stereotype – that is, happy participants formed their judgments based on stereotype and not the unimportant detailed information. Similarly, Murray et al. (1990) found that when the participant is primed to focus on differences, then positive mood increases the breadth of categories formed, which requires attention to details, compared to those in positive affect and primed to focus on similarities, where fewer, more generic categories were formed.

We would argue that, while missing or unknown inputs are particularly difficult to generate and consider, those in a positive affect may be more suited to do so either because they are using a detailed analytic process as suggested by Isen (2008) or because the internal schemas are more available thus highlighting information that is needed, but not present. Thus, in a decision situation that has missing important information, we expect to find omission sensitivity among those in positive affect and omission neglect among those in neutral affect.

## Materials and methods

### Overview of studies

Positive affect (compared to neutral) is hypothesized to increase sensitivity to missing information and, therefore, decrease product evaluations in product judgment tasks where important information is missing. In previous research, the underlying construct of omission detection has been observed using various formative indicators, including purchase interest, decreased consumer confidence, reports of information insufficiency, and lower evaluations of non-presented attributes (e.g., Sanbonmatsu et al., 1992, 1997, 2003; Kardes et al., 2006; Pfeiffer et al., 2014). As a result, when these effects are observed, consumers then tend to evaluate products less favorably, and indicate lower purchase intentions. While omission neglect can impact decision making across the spectrum (e.g., product choice, political messaging, or any type of messaging designed to change beliefs), we chose to use consumer product evaluation situation to investigate our hypotheses because product decisions are common and tend not to be polarizing. Thus, we tested this prediction across four experiments with 476 total participants, using the product choice decision and the same omission detection paradigm as this existing research.

Experiment 1 was designed to test the main effect of positive affect (compared to neutral) on product evaluations in a product choice where attribute information was present, but important attributes were missing. If our hypothesis is correct, we would expect to find those in positive affect report reduced product evaluation and lower ratings of omitted attributes (compared to neutral), but no difference on the ratings of presented attributes. This combination of results would suggest that positive affect participants (compared to neutral) are sensitive to missing information.

Experiment 2 builds on this design by adding a widely used control condition that alerts the participants to the missing attributes with a cuing manipulation (Arkes, 1991; Sanbonmatsu et al., 1992; Kardes et al., 2006). This control condition allows a test of the hypothesis that positive affect participants are naturally sensitive to missing information when processing product evaluation task where important attributes are missing. Specifically, it is hypothesized that cuing participants to attend to the missing information will have no effect on positive affect participants (because they already notice the missing information) but will increase omission detection for those in neutral affect. Further, experiment 2 uses a within-subjects design in order to enhance the robustness of the results (Hirt and Castellan, 1988), because each participant evaluates (at different time periods) multiple product stimuli in a positive affective state and a neutral affective state (counterbalanced). In addition, this study counterbalances omitted and presented attributes to rule out the alternative hypothesis that the omitted attributes are simply less liked.

Experiment 3 investigates the hypothesis that the decrease in product ratings among positive affect participants is due to a desire for more information, which is an indicator of omission detection (Pfeiffer et al., 2014). Thus, experiment 3 uses a closed-ended scale and an open-ended measure of information sufficiency to test the hypothesis that the lower reported purchase interest is driven by a desire for more information. Moreover, this experiment employs a new product category to increase the generalizability of the results.

While experiments 1, 2, and 3 were all constructed by presenting brand representations that are described with important attributes missing, experiment 4 uses a set size design to test a product description with full information against a product description with important attributes missing. Thus, we overtly test that the results are due to missing information by manipulating the amount of information presented. The results show that the hypothesized effects are limited to products with missing important information (not products described with full information). Experiment 4 also employs a new affect induction procedure as well as a new product to provide further evidence of generalizability.

## Experiment 1

### Method

#### Participants and design

Thirty-nine undergraduate students (59% female; median age 26; age range: 19–46) from a large Midwest university completed the experiment for extra course credit. Sample size was not determined by a power analysis, but rather by a simple *a priori* rule of ∼20 participants per condition. They were randomly assigned to one of two conditions of affect (positive vs. neutral). The current sample size produces an SPSS reported observed power (1−β) of 0.81 for the main effect. Thus, this sample is sufficiently powered for the analysis.

#### Procedure

Participants were told they would be participating in a market research opinion study to measure attitudes and perceptions. They were first given an affect induction task adapted from Isen et al. (1985) in which participants are presented with a word association task involving 10 words that are either positive (e.g., “beautiful,” “joy,” and “kittens”) or neutral (e.g., “house,” “pencil,” and “shoe”) in valence. This task was pretested with 22 participants from the same population from which the main sample was drawn using an independent sample *t*-test. After completing the word association task, participants in the pilot study rated their feelings on three seven-point bi-polar scales (happy/sad, pleasant/unpleasant, and good/bad; Isen and Daubman, 1984). The resulting summed scale was reliable (α = 0.84) and was significantly more positive in the positive affect group (*M* = 5.7) compared to the neutral affect group [*M* = 4.2; *t*(20) = −2.89, *p* = 0.009, η^2^ = 0.295]. A pilot study was used to evaluate the affect induction in order to reduce the possibility of alerting participants to the purpose of the word association task and the possible demand effect that would come with asking the participant to respond to the three bi-polar affect scales prior to the measurement of the constructs of interest (Perdue and Summers, 1986). Recent reviews of the literature conclude that pilot testing is more useful than including manipulation checks in the main experiments (Ejelöv and Luke, 2020), because manipulation checks may hinder hypothesis testing (Fayant et al., 2017). Thus, we use the pilot study to conclude that the affect manipulation worked as expected.

After the affect manipulation, main study respondents in both conditions were presented with a description of a fictitious pen brand labeled “Brand X,” that was ostensibly from a well-known company (stimuli adapted from Kardes and Sanbonmatsu, 1993). The pen was described by four attributes (i.e., writes smoothly, writes on a variety of surfaces, special grip for precision and control, and does not skip) and presented to the participants as “A recent ad provided the following information about brand X.” On the next page, participants rated their purchase intentions along a seven-point scale (1 = extremely unlikely to purchase to 7 = extremely likely to purchase).

After this, they were given eight attributes (the four presented attributes plus four important attributes that had not been presented – i.e., non-smear ink; durable, tungsten ball tip; guaranteed to write every time; available in a wide variety of colors) and asked to evaluate each of the eight attributes using a seven-point scale (1 = extremely bad to 7 = extremely good). The attribute ratings included in the stimulus description were averaged to form the “presented attribute rating” (α = 0.92) whereas the ratings for the four omitted attributes were averaged to form the “omitted attribute rating” (α = 0.86).

### Results and discussion

Using an independent sample *t*-test analysis, results reveal that affect predicts purchase intent [*t*(37) = 2.912, *p* = 0.006] such that those experiencing positive affect report purchase intentions that are lower by 1.2 units (on a seven-point scale) compared to those in a neutral affect state. Moreover, affect also predicts the missing attribute ratings [*t*(37) = 2.34, *p* = 0.025] such that those experiencing positive affect rated the missing attributes lower by 0.8 units (on a seven-point scale). However, the independent *t*-test is non-significant for presented attribute ratings [*t*(37) = 0.589, *p* = 0.559; *ns*] showing no statistical difference in the ratings of the presented attributes between those in positive affect and those in neutral affect (see Table 1).

**TABLE 1:** Experiment 1: Purchase intention and attribute inferences as a function of positive affect.

	Positive affect	Neutral affect
	Mean (SD)	Mean (SD)
	*N* = 20	*N* = 19
Purchase intention	3.8 (1.54)	5.0* (0.94)
Omitted attribute inferences	4.3 (1.26)	5.1* (0.92)
Presented attribute inferences	5.6 (1.24)	5.8 (0.87)

*Significantly different (using paired comparisons) from the other means in same row at *p* < 0.05.

The results from this experiment are consistent with our expectations. That is, positive affect participants may have detected that important information was missing and then, did not make simplistic inferences that missing information is consistent with the presented information. The lower ratings of the omitted attributes are hypothesized to be evidence of spontaneous omission detection such that those less favorable impressions of the missing attributes depress their purchase intentions. This result mirrors findings from Sanbonmatsu et al. (1992) where people with higher category product knowledge provided less positive product ratings (compared with those with low or moderate knowledge) when important attribute information was missing. In this case, the positive affect participants resemble those with higher category product knowledge suggesting that they also detected that important information was missing. It is important to note, however, that the hypothesized mediator (omitted attribute rating) was collected after the proposed dependent variable (purchase intention). This order of collection prohibits the overt evaluation of mediation. However, the data collection order was necessary because it has been shown that simply alerting participants about important missing information will overcome omission neglect (Arkes, 1991; Sanbonmatsu et al., 1992). Thus, we defer a mediation test to experiment 3 and 4 in which the underlying process is measured with two different observed variables. In addition, the proposed omission detection in this study is only inferred and the results do not provide direct evidence that positive affect makes one sensitive to missing information nor does it rule out the alternative hypothesis that the presented attributes were simply more positive compared to the omitted attributes. Experiment 2 was designed to directly address these issues by including a control condition.

## Experiment 2

People are usually not sensitive to missing information unless they are provided with a cue or signal that certain information is missing (Arkes, 1991). Such a cue focuses attention to the specific missing attributes, causes observers to desire that missing information (Sanbonmatsu et al., 1992), and then causes them to adjust their evaluations to accommodate the unknown elements. To determine if the results of experiment 1 are due to attention to the missing information among those in positive affect, experiment 2 adds a control condition that alerts some participants to the omitted attributes. In this way, the design provides a control condition in which participants are made aware of the missing information to serve as a comparison. That is, after reviewing the product stimuli, those in the cuing condition are asked to rate all product attributes (presented and omitted) before providing an overall purchase intention. This manipulation has been used in the past to successfully draw attention to the omitted information (Sanbonmatsu et al., 1992; Kardes et al., 2006). We expect that if positive affect spontaneously increases participants’ sensitivity to omissions, the cuing manipulation should have little additional effect on positive affect participant product evaluations and purchase intentions. In contrast, cuing should have a significant effect for those in the neutral affect state. Thus, we expect the neutral non-cued results to differ from the results of the remaining three conditions. This pattern of results indicates that positive affect participants naturally detect the omissions so cuing has no effect, but the neutral affect participants naturally neglect the missing information so cuing has an effect by causing omission detection.

### Method

#### Participants

One hundred thirteen undergraduate introductory business students from a large Midwest university participated in the first session of the experiment. Of these, 93 (30% female, M_*age*_ = 22) were also present 1 week later and constituted the final sample. Sample size was not determined by a power analysis, but rather a simple *a priori* rule of ∼20 participants per condition. A *post hoc* estimate of power was executed using the SPSS repeated measures ANOVA calculation of observed power and effect size. The results show that current sample size is sufficiently powered for the observed effect sizes (η^2^ = 0.08 and η^2^ = 0.13) with a power (1−β) of 0.76 and a power (1−β) of 0.94 for the between- and within-subjects analyses, respectively.

#### Design

Participants in this experiment took part in two experimental sessions that were run 1 week apart. They were randomly assigned to either cuing or no-cuing conditions, which was the same in both sessions. However, the combination of positive affect and product type (a pen vs. a calculator) varied in a Latin square design so that participants who were exposed to a given combination of positive affect and product type in one session were exposed to the opposite combination of these variables in the second session.

Specifically, eight groups of participants were constructed. Four groups were cued to the missing information and four groups were not cued. Within each cuing condition, one group evaluated a pen after being induced to experience positive affect. A second group evaluated a calculator in this affect condition. The other two groups rated the products under neutral affect. Then, in the second session, participants took part in the same cuing condition as Session 1, but the product they rated and the affect they experienced were the opposite of those to which they were exposed in Session 1. See Supplementary Appendix A for a description of the design and pooling test. All products were presented with a short list of attributes consistent with the presentation used in experiment 1.

#### Stimulus attributes

Each product was described by four presented and four omitted attributes, which were counterbalanced by set within each experimental condition. This design will control for favorability of the presented and omitted attributes since each set of attributes will serve each role. The pen descriptions were the same as those described in Study 1 except that the omitted and presented attribute sets were counterbalanced. The calculator attributes were adapted from Mantel and Kardes (1999) and presented in a similar format. The two sets of four attributes associated with the calculator were either (a) “automatic shut-off to preserve the battery,” “unit is small and compact,” “easy to program,” and “has many features” or (b) “very durable,” “battery lasts a long time,” “output is easy to read,” and “keys are silent when pressed”). This counterbalancing technique was employed to test whether effects were due the content of the attributes presented (i.e., that the omitted attributes were inherently less liked) rather than number of attributes presented.

#### Procedure

Participants first performed the word association task employed in experiment one to induce either positive or neutral affect. Participants then reviewed information about four attributes of the target product (the pen or the calculator). Then, in non-cued conditions, they answered the same purchase intention questions administered in the first study and rated both presented and omitted attributes of the product they had considered. In cued conditions, however, they rated all eight attributes (four presented and four omitted) before reporting purchase intentions. The procedure in Session 2 was the same except that the product and affect were counterbalanced as indicated earlier.

Finally, participants provided demographic information and responded to two questions designed to assess insight into the mood manipulation. No participants guessed that the word association task related to mood or affect. In addition, in response to the prompt to recall items from the previous week word association task, several respondents tried to recall words from “last week,” but no one successfully did so.

#### Manipulation check

The effectiveness of the affect manipulation was confirmed based on data from 46 participants from a pilot test pulled from the same population. These pilot participants reported their affective reactions immediately after performing the word association task by responding to the three bipolar scales used in the first experiment. This pilot test method of manipulation check is used in order to minimize the possible demand effect of alerting the participants of the purpose of the word association task (Perdue and Summers, 1986; Kardes et al., 2006; Fayant et al., 2017; Hauser et al., 2018). As with the first experiment, the manipulation check was successful [*F*(1,44) = 12.58, *p* < 0.001, η^2^ = 0.22; neutral affect, *M* = 4.7; positive affect, *M* = 5.9].

### Results and discussion

Results were analyzed using a repeated measures ANOVA as a function of cuing conditions, affect, product type and experimental session. There were no main or interactive effects for product type suggesting that all analyses can be pooled across product type. For simplicity, we report the conceptually meaningful main and interaction effects that emerged from the repeated measures ANOVA after pooling as well as the relevant planned comparison run within the ANOVA to control for error across multiple comparisons. A few comparisons were run with *post hoc* independent *t*-tests and are indicated as such.

**FIGURE 1:**
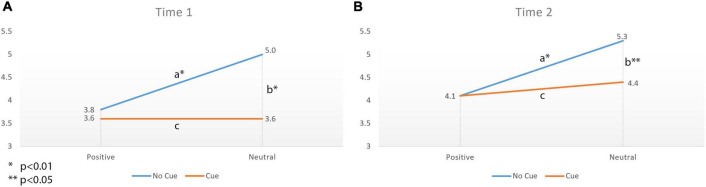
Experiment 2: Purchase intention as a function of positive affect and omission cuing at **(A)** Time 1 and **(B)** Time 2. Note: the repeated measures analysis shows a significant difference at both Time 1 and Time 2 between Neutral-No-Cue condition and Positive-No-Cue condition (a) as well as between Neutral-No-Cue condition and Neutral-Cued condition (b), but no significant difference between the two cued conditions (c).

Product evaluations pooled over product type are summarized in Table 2 as a function of cuing, affect condition, and session. Analyses yielded a three-way interaction of cuing by affect by experimental session [*F*(1,89) = 7.20, *p* = 0.01, η^2^ = 0.08]. As expected, positive affect participants, in the absence of cuing, reported purchase intentions that are 1.2 units lower (on a seven-point scale) compared to neutral affect participants when comparing within session [Session 1: *F*(1,89) = 7.35 *p* = 0.01, η^2^ = 0.08; Session 2: *F*(1,89) = 8.42, *p* = 0.01, η^2^ = 0.09]. However, when they were alerted to the missing information (i.e., cued condition – they rated attributes before reporting purchase intentions), the purchase intentions were low and did not depend on affect condition with cross condition differences of less than 0.1 (on a seven-point scale) during session 1 and less than 0.3 on the same scale during session 2 (planned contrast *p* > 0.48). A *post hoc* independent *t*-test was used to compare the cued condition (where participants rated the attributes prior to assessing purchase intent) to the condition in which omission neglect was expected (Neutral non-cued, see Figure 1). As expected, the observed differences were significant – 1.4 units (on a seven-point scale) in session 1 [*t*(72) = −3.98, *p* < 0.001] and 1.1 units on the same scale in session 2 [*t*(63) = −2.873, *p* = 0.01]. In addition, the cued condition reported only minimal difference on purchase intention compared the condition where omission detection was expected (Positive non-cued) with only 0.2 units difference in session 1 [*t*(63) = −0.52, *p* = 0.60, *ns*] and 0.1 units difference in session 2 [*t*(72) = 0.48, *p* = 0.63, *ns*]. These data show that, as expected, the purchase intentions for non-cued participants who were in neutral affect were significantly higher than those in cued condition as well as those non-cued participants who were in positive affect.

**TABLE 2:** Experiment 2: Purchase intention and omitted attribute inferences as a function of positive affect and omission cuing.

	Positive affect		Neutral affect	
	Mean (SD)		Mean (SD)	
	No Cuing^1^	Cuing^2^	No Cuing^1^	Cuing^2^
	*N* = 19_T1_, 28_T2_	*N* = 23_T1_, 23_T2_	*N* = 28_T1_, 19_T2_	*N* = 23_T1_, 23_T2_
Purchase intention (T1)	3.8 (1.47)	3.6 (1.20)	5.0* (1.53)	3.6 (1.59)
Purchase intention (T2)	4.1 (1.58)	4.1 (1.50)	5.3* (1.37)	4.4 (1.23)
Omitted attribute inferences (T1)	4.0 (1.66)	3.6 (1.39)	5.1* (1.46)	4.0 (1.20)
Omitted attribute inferences (T2)	4.1 (1.75)	4.4 (1.1)	5.0* (1.44)	4.2 (0.86)

*Significantly different (using paired comparisons) from the other means in same row at *p* < 0.05.

^1^The “No Cuing” condition was executed by asking the participant to provide product evaluation directly after viewing the stimulus.

^2^The “Cuing” condition was executed by asking the participant to evaluate present and missing attributes prior to providing product evaluation (signaling missing attributes).

In addition, the analysis of the omitted attribute ratings is also consistent with the hypotheses. That is, the repeated measures ANOVA revealed a significant three-way interaction of cue by affect by experimental session [*F*(1,89) = 5.54, *p* = 0.02, η^2^ = 0.06]. For those who were not cued, positive affect decreased evaluations of omitted attributes compared to neutral affect by 1.1 units (on a seven-point scale) at session 1 [*F*(1,89) = 6.35, *p* = 0.01, η^2^ = 0.07] and 0.9 units at session 2 [*F*(1,89) = 4.52, *p* = 0.04, η^2^ = 0.05, see Table 2]. However, the perceptions of omitted attributes did not significantly differ across affect conditions when participants were cued [a difference of 0.3 units at session 1 (*p* = 0.34, *ns*); 0.2 units at session 2 (*p* = 0.61, *ns*); on a seven-point scale]. A *post hoc* independent *t*-test was used to compare the condition where participants rated the attributes prior to assessing purchase intent (Cued condition) to the condition in which omission neglect was expected (Neutral non-cued). Here, neutral non-cue participants rated the omitted attributes more positively than those in the cued condition [1.3 units higher at session 1, *t*(72) = −3.82, *p* < 0.001; and 0.7 units higher at session 2, *t*(63) = −2.30, *p* = 0.03]. In addition, the condition where omission detection was expected (Positive non-cued) the ratings of omitted attributes did not significantly differ from the cued condition (0.2 scale points at both session 1 and 2, *p* = 0.65, *ns*). These data show that, as expected, the ratings for the omitted attributes for non-cued participants who were in neutral affect were significantly higher than those in cued condition and those in positive affect and not cued. Taken together, both analyses suggest that those in positive affect may have noticed the missing information even without the cue.

These results provide further evidence that positive affect enables participants to both notice that important information is missing, and to moderate their purchase intentions accordingly. Even when controlling for the content and favorability of omitted attributes by counterbalancing the attribute sets, in the absence of cuing, positive affect participants evaluated omitted information less favorably and reported lower purchase intentions than those in neutral affect. When participants were sensitized to the omitted attributes (cued), however, they evaluated omitted attributes unfavorably and reported lower intentions to purchase regardless of the affect they were experiencing. Thus, positive affect and cuing had similar effects. It is important to note that this relationship, while consistent with the omission neglect literature, is an inferred rather than a measured relationship. In experiment 3, we further investigate the process by testing proposed mediators.

## Experiment 3

Existing research on omission detection has shown that people temper their evaluations of products when they notice limited information is provided and they want additional information about the target product (Sanbonmatsu et al., 1997). Thus, experiment 3 builds on the previous two studies by including an overt measure of the proposed mediator (i.e., need for additional information) and a qualitative description of the decision process. Moreover, our qualitative data serve as an indication of the thought process used by participants.

### Method

#### Participants

One hundred thirty-two undergraduate introductory business students (55% female, M_*age*_ = 21 years) from a large Midwest university participated for extra course credit. They were assigned to cells of a 2 (affect: positive vs. neutral) x 2 (cued vs. not cued) between-subjects design. An *a priori* power analysis using G*Power 3.1 (Faul et al., 2009) recommends a sample size of 128 for this analytical design and a medium effect size. The resulting sample appears to be sufficient given the observed effect sizes of between η^2^ = 0.06 and η^2^ = 0.12 and observed power (1−β) of between 0.82 and 0.96 for the relevant comparison contrasts of interest on the dependent variable (purchase interest) and the proposed mediator (desire for additional information).

#### Design and manipulation check

Participants completed the word association task employed in Study 1 to induce either positive or neutral affect. The effectiveness of the manipulation was confirmed based on data from 47 participants from a holdout sample (pulled from the same population) who reported their affective reactions immediately after performing this task along the three bipolar scales used the first experiment. As with the first two experiments, the manipulation check was successful [*F*(1,45) = 5.64, *p* = 0.02, η^2^ = 0.11; neutral affect, *M* = 5.2; positive affect, *M* = 5.9]. Therefore, a manipulation check was omitted from the main experiment to reduce demand effects, disruption, and other artifacts that may occur when affect is measured along with the primary tasks in the main experiment (Perdue and Summers, 1986; Fayant et al., 2017; Ejelöv and Luke, 2020).

After the affect manipulation, participants were asked to imagine they were planning a vacation they would take in a few months. Participants were then given a description of a vacation, purportedly extracted from an advertisement for a possible vacation available for purchase. The vacation package described five attributes (destination: Cancun, Mexico; hotel rating: ^****^; ocean view; free internet access; 24-h fitness facility). Price was specifically left off the list of attributes because consumers tend to infer information about the product when they have a relative price. For example, consumers infer that a higher priced product is healthier (Haws et al., 2017), and of higher quality (Cronley et al., 2005).

This study used the same cuing procedure as in experiment 2. Participants in cued conditions were asked before evaluating the vacation to rate both the five presented attributes and four missing attributes. Participants in no-cuing conditions evaluated the vacation first. Specifically, they were asked, “if you were planning to take a vacation of this type, how likely are you to choose to go to the destination as described rather than some other destination?” and responded along a scale from 1 (extremely unlikely) to 7 (extremely likely).

Then participants were asked to “describe in as much detail as you can how you went about evaluating the vacation package” followed by a full page of lines to encourage full descriptions. These comments were read by two coders who were naïve to the study design. The coders were provided a list of presented attributes and asked to group each participant’s response into three categories summarizing the tone of the comments: (1) simple focus on presented attributes, (2) elaboration on presented attributes, and (3) concerns or requests for more information. An interrater reliability analysis using the Kappa statistic was performed to determine the consistency among raters. The raters show substantial agreement (Kappa = 0.75) and all disagreements were resolved through discussion.

To assess the need for additional information, participants reported the extent to which they wished they had more information along a scale from 1 (no more information needed) to 7 (more information needed). To make this focal question less noticeable, it was presented as the sixth question within a set of eight bi-polar items that were related to their confidence in their decision.

### Results

The results of the previous experiments were replicated with a moderation analysis using 5000 bootstrap samples for bias corrected 95% confidence intervals using Hayes (2017) model 1. The interaction of cued manipulation and affect significantly predicts purchase intention [*B* = 0.91, SE = 0.46, *t*(128) = 1.96, *p* = 0.05] such that the IV, affect, significantly predicts the DV, purchase intent, when participants are non-cued[*B* = −0.97, SE = 0.33, *t*(128) = −2.91, *p* < 0.01], but not when participants are cued [*t*(128) = −0.18, *p* = 0.86, *ns*]. Planned contrasts run within the ANOVA showed the expected patterns for the dependent variable and the proposed mediator (see Table 3). That is, neutral affect participants who were not cued to the missing information reported a significantly higher purchase intention [+0.9 scale points on seven-point scale, *F*(1,128) = 8.48, *p* < 0.01, η^2^ = 0.06] and a significantly lower need for additional information [−1.1 on a seven-point scale, *F*(1,128) = 9.84, *p* < 0.01, η^2^ = 0.07] compared to the condition in which participants are hypothesized to be aware of the missing information (i.e., those in positive affect who were not cued). Similarly, neutral affect- non-cued also report higher purchase intent [+1.2 scale points, *F*(1,128) = 14.33, *p* < 0.01, η^2^ = 0.10] and a lower need for additional information [−1.4 scale points, *F*(1,128) = 17.19, *p* < 0.01, η^2^ = 0.12] (see Table 3) compared to neutral affect-cued condition. Finally, the contrasts show there were no statistical differences (*p* > 0.34) in the three groups hypothesized or known to be aware of the missing information (i.e., positive affect non-cued (hypothesized) and cued participants regardless of affect state (manipulated to be aware of the missing information).

**TABLE 3:** Experiment 3: Purchase intention and omitted attribute inferences as a function of positive affect and omission cuing.

	Positive affect Mean (SD)		Neutral affect Mean (SD)	
	No Cuing^1^	Cuing^2^	No Cuing^1^	Cuing^2^
	*N* = 31	*N* = 34	*N* = 33	*N* = 34
Purchase intention	5.0 (1.56)	4.7 (1.15)	5.9* (1.25)	4.7 (1.36)
Perceived need for additional information	5.6 (1.56)	5.7 (1.45)	4.5* (1.64)	5.9 (1.04)
Omitted attribute inferences	3.9 (1.18)	4.3 (1.08)	4.6* (1.20)	3.9 (1.21)

*Significantly different (using paired comparisons) from the other means in same row at *p* < 0.05.

^1^The “No Cuing” condition was executed by asking the participant to provide product evaluation directly after viewing the stimulus.

^2^The “Cuing” condition was executed by asking the participant to evaluate present and missing attributes prior to providing product evaluation (signaling missing attributes).

#### Mediation

**FIGURE 2:**
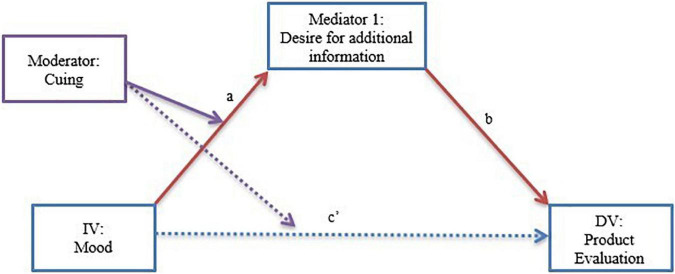
Experiment 3: Moderated mediation (Model 8, Hayes, 2017) the effects of cuing interaction on the relationship between mood and product evaluation through desire for additional information. Note: the analysis shows a significant path through desire for additional information (a → b, *p* < 0.05) and no direct path (c′, *ns*).

The proposed mediators are collected after the IV, but participants were asked to answer the question thinking about the decision process. Analyses to evaluate temporal order implications were tested and can be reviewed in Supplementary Appendix B. The model presented herein is the hypothesized model and also has the best fit. That is, the proposed mediator (desire for additional information) is considered using Hayes (2017) bootstrapping procedure for moderated mediation (Model 8, see Figure 2). The results show that the overall model is significant (*p* < 0.01). The indirect effect is significant for the non-cued condition (Effect = −0.27, SE = 0.13, LLCI = −0.55, ULCI = −0.06) but not in the cued condition (the confidence interval contains 0: LLCI = −0.10, ULCI = 0.22, *ns*). Further, the index of moderated mediation shows that there is a significant different between the two conditional indirect effects (Index = 0.33, SE = 0.15, LLCI = 0.06, ULCI = 0.64). Therefore, we can conclude that omission detection is present given that desire for additional information is an important mediator of this process (Preacher and Hayes, 2008). In addition, follow-up correlations show that for those in positive affect, the effect is driven by the feeling of information insufficiency. That is, purchase intent for those in positive affect (but not for neutral affect) is correlated with need for additional information (see Supplementary Appendix B). Taken together, the mediation analysis and the correlations, suggest that those in positive affect adjust their purchase intent because they have a feeling that information is missing.

#### Qualitative decision process

To more completely understand how the decision process differs across affect groups, we looked at the open-ended descriptions of the process among the 63 participants who were not cued to the missing information and provided a response to the process question. We excluded those that were cued since the cue itself may influence the content of the process description. As described above, the coded responses were tallied across the two affect groups and the three classifications of comments (i.e., focus on presented attributes, elaboration on presented attributes, and request for additional information). The pattern of results differed across the six categories [χ^2^(2, *N* = 63) = 15.66, *p* < 0.001].

(1) Simple focus on the presented attributes (e.g., “It was a four-star hotel with an excellent view of the ocean. There was wireless internet and 24-h workout facility” male). N_positive_ = 6; N_neutral_ = 23 [χ^2^(1, *N* = 63) = 8.43, *p* < 0.01].
(2) Elaboration based on presented attributes (e.g., “Being in Cancun with an oceanfront view, I picture it being very relaxing and a good escape from reality. I also imagine all the tourist attractions in the area.” male). N_positive_ = 11; N_neutral_ = 5 [χ^2^(1, *N* = 63) = 2.86, *p* = 0.09].
(3) Concerns or requests for more information (e.g., “I did not, however, give a higher rating because food was not mentioned in the package. I have no idea how pricey food is or if it is all-inclusive” female). N_positive_ = 13; N_neutral_ = 5 [χ^2^(1, *N* = 63) = 4.37, *p* = 0.04].

This analysis of the qualitative data provides further evidence that positive affect participants are more likely to notice missing information and use that knowledge in the product evaluation. The data show that twice as many positive affect participants (13) requested additional information compared to neutral affect participants (5). Conversely, four times as many neutral affect participants (23) listed the presented attributes without elaboration compared to their positive affect counterparts (6). Finally, some mentioned the presented attributes but gave those attributes greater meaning; this was slightly more likely among positive affect participants.

### Discussion

The results from experiment three replicate the results from the first two experiments. Using an overt measure of the perceived need for additional information, experiment three shows that this need mediates the relationship between affect and product choice when information is limited. The qualitative analysis of the open-ended comments further supports the conclusion that individuals in a positive affective state thought more carefully about the specific details of the product description, detected omissions in the product description, and desired additional information to make an evaluation of the product. Likewise, positive affect (compared to neutral affect) participants elaborated on the product information presented, which may suggest that the participant is comparing the description to information from the general product category (vacations) and identifying important items that are missing. This pattern of results is consistent with omission sensitivity for those in positive affect and omission neglect for those in neutral affect.

## Experiment 4

Experiments 1–3 show that positive affect individuals (compared to neutral) are more likely to indicate a need for additional information and moderate their purchase interest accordingly when they evaluate a product described without important information. Conversely, neutral affect individuals only moderate their purchase interest when they are overtly alerted to the missing information. While this pattern of results is consistent with the omission neglect literature and suggests that positive affect increases sensitivity to omissions compared to their neutral affect counterparts, a more direct test of our hypothesis would be the use of a set size manipulation. In experiment 4, the product description will be either a missing information condition or a full information condition. Specifically, we hypothesize that positive affect will produce sensitivity to omissions when the product is described with fewer attributes and missing important information. Conversely, positive affect participants evaluating the product with full information and neutral affect participants regardless of the completeness of the description will not be sensitive to any missing information either because full information is present or because of omission neglect (neutral affect missing information condition).

### Method

#### Pilot studies for stimuli creation

To create stimuli that would differ in the completeness of the attributes conveyed, a restaurant was used as the target. Totally 119 Mturk participants were asked to rate 32 attributes of a restaurant in terms of importance and valence. Next, the completeness of the information was investigated using 104 Mturk participants. Specifically, each participant was presented with the attributes one at a time in the order of their importance (as rated by the first pilot). After each attribute, participants were asked whether they wanted additional information or were ready to evaluate the restaurant. The 84% of the participants indicated readiness to evaluate the restaurant before receiving the 13th attribute whereas 60% of the participants continued to want additional information even after seeing the fifth attribute. Thus, it was concluded that a stimulus described by the 13 most important attributes would represents full information, and a stimulus 5 attributes of lower importance would represent missing information (see Supplementary Appendix C).

Finally, the stimuli were verified in a third pre-test of 47 Mturk participants. Participants were presented with one of the stimuli executions full information (13 attributes) or missing important information (5 attributes) and a decision scenario (e.g., imagine as part of your job, you are choosing a restaurant for your boss and a top client). After viewing the assigned restaurant stimulus, each participant was asked the extent to which each attribute describes the evaluated restaurant on a scale from 1 (Strongly Disagree) to 5 (Strongly Agree). For each attribute that was missing from the partial information version, there was a significant difference in reported agreement across the two conditions (*p* < 0.01) and for each attribute that was included in both descriptions there was no difference in reported agreement (all pared comparisons are non-significant). This indicates that none of the omitted attributes were easily inferred from the presented attributes in the missing attribute stimuli.

#### Participants and design

Participants were 192 MTurk workers (56% female; M_*age*_ = 33 years). They were assigned to cells of a 2 (affect: positive vs. neutral) x 2 (Information completeness: complete vs. incomplete) between-subjects design. An *a priori* power analysis using G*Power 3.1 (Faul et al., 2009) recommends a sample size of 183 for this analytical design and a medium effect size. The *post hoc* sensitivity measures also support the sample size to be sufficiently powered for this analysis. The resulting sample shows an observed effect sizes (η^2^ = 0.04 and η^2^ = 0.03) and observed power (1−β) (0.78 and 0.65) for the relevant contrasts of interest on the dependent variable (purchase interest) and the proposed mediator (relative thought focus), respectively.

#### Procedure

To increase the robustness of our findings, we used a different affect induction task in experiment 4. We chose the autobiographical story/imagery method, because this method has been employed in numerous similar consumer decision-making studies (e.g., Schwarz and Clore, 1983). Here, participants who were assigned to the positive affect condition were given 5 min to write a story about “a situation that happened to you and that made you feel really happy/joyful.” If they finished writing before the 5 min expired, they were instructed to “please re-read your story and remember the feelings of that time.” The online survey program would not allow the participant to advance to the next question until the end of the 5-min period. Participants in the neutral affect condition were given 5 min to write a story about “a time you went to the grocery store.” Similar instructions have been used to induce a neutral state that is significantly different from positive (e.g., Grawitch et al., 2003). As in the positive affect condition, participants were instructed to re-read their story and were not allowed to advance until the 5-min period was complete.

As a manipulation check, participants were asked to report their current feelings by selecting a box on a 2-dimensional grid (Affect grid; Russell et al., 1989) in which the horizontal dimension ranged from 1 (unpleasant) to 9 (pleasant) and the vertical dimension pertained to arousal (1 = no arousal, 9 = high arousal). Participants were told to choose any box on the grid to indicate the “exact shade and intensity” of their current mood. In this experiment, we chose to include the manipulation check within the main study for two reasons. First, we wanted to rule out motivational intensity (or arousal) as an alternate explanation for the findings and show that the manipulations controlled for arousal across cells (Harmon-Jones et al., 2013; Domachowska et al., 2016). Second, the grid measure was considered less obtrusive given the inclusion of both affect and arousal within one selection task as compared to the three separate items on the bi-polar affect scales used in the first three experiments (Russell et al., 1989; Hauser et al., 2018). Thus, this manipulation check was included for all participants immediately after the mood induction task. As in the first three experiments, participants reported feeling more positive in the positive affect condition (*M* = 7.28) than in the neutral affect (*M* = 6.44) condition [*F*(190) = 9.17, *p* < 0.01]. As expected, differences in arousal were not significant [*F*(190) = 2.93, *p* = 0.09, *ns*; neutral affect, *M* = 4.88; positive affect, *M* = 5.39].

Next, participants were told to imagine that they were “in charge of planning a dinner at a local restaurant for your boss and a top client.” The restaurant was portrayed as “recommended by a friend” and described by a list of either 13 attributes (in full-information conditions) or 5 attributes (in missing-information conditions), based on the pretesting described earlier. This presentation of the product replicates the procedure used in experiments 1–3.

Participants rated the likelihood of either choosing the restaurant or postponing the decision and looking for other options” along a seven-point scale from 1 (extremely likely to postpone) to 7 (extremely likely to choose). This item, then, is used to represents purchase interest.

To measure relative thought focus, participants were asked immediately after their decision to report “in as much detail as you can how you went about evaluating the restaurant.” The responses were read by two naïve raters who coded them as either 1 (focused on presented information (what you see is all there is); e.g., “It looked like a busy restaurant,” “The decor livened up the place”) or 2 (focused on non-presented or missing information (analytic or extended thinking); e.g., “there was not much info about the restaurant… I would look at other options that might describe their location better,” “no idea how the food is either without any customer ratings”). Raters were provided with a list of presented attributes for each condition and trained to properly code some sample items prior to independently coding the participants’ responses. An interrater reliability analysis using the Kappa statistic was performed to determine the consistency among raters. The raters show substantial agreement with a Kappa = 0.73 (*p* < 0.001) and differences were resolved through discussion. The difference in the number of presented, unelaborated thought items and the number of non-presented, elaborated thought items was used to indicate the relative disposition of the participant to focus on presented information. (Note: High values represent more thoughts focused on presented information and low values represent more thoughts focused on non-presented, elaborated information).

### Results

Evaluations are shown in Table 4 as a function of affect, and information completeness. As hypothesized, the analyses of the purchase interest measure yielded an interaction between affect and information completeness [*F*(1,188) = 5.1, *p* = 0.03, η^2^ = 0.03].

**TABLE 4:** Experiment 4: Product evaluation and relative thought focus as a function of positive affect, task type, and information completeness.

	Positive affect Mean (SD)		Neutral affect Mean (SD)	
	Full information	Missing information	Full information	Missing information
	*N* = 44	*N* = 54	*N* = 55	*N* = 39
Purchase interest	5.3 (1.25)	4.7^a^ (1.43)	5.2 (1.29)	5.5 (1.17)
Relative thought focus (higher numbers = focus on presented informaiton; lower number = focuson on non-presented information)	2.2 (2.54)	0.7^b^ (1.82)	1.9 (1.88)	1.6 (1.46)

^a^Significantly different (using planned contrast within ANOVA) from the other means in same row at *p* < 0.01.

^b^Significantly different (using planned contrast within ANOVA) from the other means in same row at *p* < 0.02.

The results of the planned comparison run within the ANOVA suggested that in the missing-information condition, those in a positive affect reported significantly lower purchase interest compared to those in a neutral affect [−1.2 scale points on a seven-point scale, *F*(188) = 7.50, *p* < 0.01, η^2^ = 0.04]. Further, in the positive affect condition those presented with the missing-information stimulus reported significantly lower purchase interest compared to those in positive affect and presented with the full-information stimulus [−0.6 scale points on seven-point scale, *F*(188) = 4.08, *p* = 0.05, η^2^ = 0.02]. There is no significant difference in the purchase interest between those in the positive affect condition evaluating a product with full information and those in the neutral affect condition evaluating a product with full information (−0.1 scale points, *p* = 0.68, ns) or the those in neutral affect condition evaluating a product with missing information (+0.2 scale points, *p* = 0.42, *ns*). Taken together, these results are consistent with omission detection among those in positive affect, and omission neglect among those in neutral affect (i.e., those in neutral affect evaluate the missing information stimuli as if they had full information). Further, these results replicate the findings of experiments 1–3.

**FIGURE 3:**
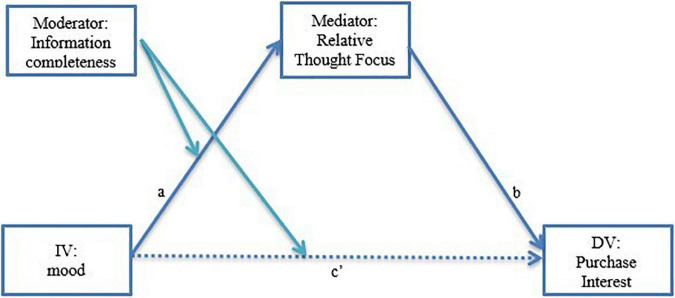
Experiment 4: Moderated mediation (Model 8, Hayes, 2017) testing the effects of the mood on product evaluation through relative thought focus moderated by information completeness. Note: the model shows a significant conditional indirect effect (a,b) through relative thought focus only when information is missing (path a → b, *p* < 0.05; path c, *ns*); when information is complete: path a → b, *ns.* The index of moderated mediation (Index = 0.27) shows that the conditional indirect effects are significantly different (*p* < 0.05) across the two information completeness conditions.

To test the mediation hypothesis, a full moderated mediation using ordinary least squares path analysis with bias-correcting bootstrapping using 5000 re-samples and 95% confidence interval was run using Hayes (2017) model 8. This model tests the hypothesis that the influence of affect (IV) on purchase interest (DV) is moderated by information completeness and conditionally mediated by the relative focus of the thoughts (i.e., focus on presented information vs. focus on non-presented information) during the decision process (see Figure 3). The results show that the model is significant (*p* < 0.001) and there is a conditional indirect effect (a,b) through the mediator (i.e., relative thought focus) only when information is missing (Effect = 0.41, SE = 0.154, LLCI = −0.732. ULCI = −0.125), but not in the full information condition where the indirect effect is non-significant (LLCI = −0.248, ULCI = 0.495, *ns*). Further, the index of moderated mediation is significant (Index = 0.544, SE = 0.232, LLCI = 0.088, ULCI = 1.00) suggesting that there is a meaningful difference in the two paths. Finally, the direct effect (c′) has a confidence interval including 0 and is also non-significant (LLCI = −0.275, ULCI = 0.900, *p* = 0.29, *ns*) when the mediator is included in the model. These results support the expectation that those in a positive affective state are more likely to notice missing information and moderate their purchase intent when they are evaluating a product with missing important information.

While experiments 1–3 used a manipulation to alert participants of the missing information, experiment 4 overtly examines the difference between product evaluation under partial and full information. That is, positive affect participants report more moderate product evaluations when viewing the missing-information stimulus compared to those in a neutral affect state viewing missing information stimulus and compared to both full-information conditions. The mediation analysis shows that when the stimulus has missing information, the greater focus on thoughts related to non-presented attributes mediates the relationship, supporting the theory that positive affect stimulates individuals to focus on missing information by identifying the important information that is missing and moderating their purchase intention as a result.

## A test of convergence

To provide additional support for this empirical package, we conducted two internal meta-analyses to measure the overall reliability of our results across the four experiments. Specifically, we show that, when presented with a product described with missing important information and not alerted to that missing information, positive affect individuals are more likely (compared to neutral affect) to perceive a need for additional information (experiment 3) and produce thoughts that were more focused on missing information (experiment 4). Although each experiment used a different measurement tool, the two variables were standardized into comparable measures of the same underlying construct and analyzed using SPSS meta-analysis. The results indicate a medium effect size across the two studies (Cohen’s *d* = −0.63; *Z* = −3.82, *p* < 0.001; CI: −0.95, −0.31). Importantly, the results of the test for homogeneity/heterogeneity produce a non-significant *Q* and an *I*^2^ that is less than 25%. Taken together, these results reveal that meaningful variance does not exist (*Q* = 0.14, df = 1, *p* = 0.71, *I*^2^ = 0.00). Additionally, we investigated the convergence of the dependent variable (purchase interest) across the five data collections – that is, both time periods of the repeated measures study (experiment 2) and the remaining three between subjects designs (experiments 1, 3, and 4). The results of the meta-analysis indicated a medium size effect across the four studies (Cohen’s *d* = −0.72; *Z* = −5.84, *p* < 0.001; CI: −0.96, −0.48) and no meaningful variance across the studies (*Q* = 0.93, df = 4, *p* = 0.92; *I*^2^ = 0.00). These two meta-analyses provide direct support for the claim that this package of four experiments show consistent results of the underlying hypothesized constructs and support the hypothesis that positive affect participants are more likely to notice important missing information and evaluate the described product through that lens.

## General discussion

Collectively, the results of four experiments support the prediction that when people experience positive affect, they are more sensitive to missing information in the product description or advertisements, which leads to lower purchase intent if important information is, in fact, missing. Sensitivity to omissions occurs because consumers recognize that important information is missing from the product description and they do not feel confident enough to make superficial inferences based on the information available to them (Kardes et al., 2006). While this sensitivity to missing information is often difficult to capture, in the current studies, it was inferred (experiments 1, 2, and 3) using a cuing manipulation that is standard in omission neglect literature (Sanbonmatsu et al., 1992; Kardes et al., 2006) and by comparing a “full information” stimulus vs. a “partial information” stimulus (experiment 4). The latent variable of underlying processing strategy is estimated with the measure of (a) greater attentional focus on non-presented information (experiment 4), (b) an increase in perceived information insufficiency (experiment 3), and (c) via the historic proposed indicator (Kardes et al., 2006) of lower ratings of the non-presented attributes of the product (experiments 1, 2, and 3). As a latent variable, it is important to note that the measurement of the underlying processing strategy is never perfect. However, by including three different measures of this underlying variable (all of which produce the same relationships) we can feel more confident that the triangulated results are more informative than if we had used any one measure by itself.

Specifically, experiment 1 showed that positive affect decreased evaluative judgment extremity, relative to neutral affect control participants and hypothesized that this result was driven by a sensitivity to missing information. Experiment 2 extended these results by providing a cue or signal to some participants that certain information is missing, thus covertly alerting some participants to missing information. If positive affect increases sensitivity to omissions (as hypothesized), we expected that it would decrease participants’ product evaluations and purchase intentions even in the absence of cuing. Cuing should, therefore, have little additional effect on positive affect participants but significant effect on neutral affect participant. Results were consistent with these expectations. Experiment 3 tested an overt measure of need for information as the mediating mechanisms that may drive the relationship between affect and lower product evaluations. In this experiment, participants rated their need for information after evaluating a product with important information missing. Individuals in positive affect reported a higher need for additional information, and that increased need for additional information mediated the relationship between affect and purchase intentions. Finally, experiment 4 uses stimuli that had either full information or partial (missing) information to confirm the effects found in the first three studies. This study shows that when people are making a purchase decision with missing information, positive affect increases the likelihood one will report more thoughts related to the missing information (i.e., focused on non-presented or missing information), which results in less positive product evaluations and purchase intentions – that is, they are sensitive to missing information (Kardes et al., 2006, 2022).

Taken together, these four experiments show that positive affect produces differential results from those in a neutral affect such that those in positive affect look beyond the stated information (WYSIATI, or What You See Is All There Is; Kahneman, 2011) to mention thoughts focused on non-presented or elaborated information. Further, when evaluating a product with incomplete information, positive affect participants (but not neutral affect participants) rate the available information as less sufficient (experiment 3) and report more moderate purchase intentions (experiments 1, 2, 3, and 4) suggesting that positive affect participants are more sensitive to omissions.

### Theoretical implications

These results have implications for our theoretical understanding of how positive affect impacts information processing. Our results align with the viewpoint that the effects of positive affect allow participants to critically evaluate presented information (Isen, 2008) and are consistent with the idea that internal schemas or script may be activated highlighting the information that is missing (Bless et al., 1996). Our data is inconsistent with the hypothesis that positive affect facilitates heuristic processing and participants would use their mood as a heuristic cue to rate a product more favorably (i.e., mood congruence; Bodenhausen, 1993; Gorn et al., 1993; Martin et al., 1993; Bodenhausen et al., 1994; Pham, 1998). Our data shows the opposite – thus supporting the improved information processing hypothesis. Although this process was tested in the context of presented attributes associated with a product, it could have implications into other situations where it is important for information to be evaluated for completeness prior to making a decision.

The current results help our understanding of how people evaluate products when important information is missing and builds on previous research that shows environmental cues as well as an individual’s category expertise can alert people to notice that an advertisement is missing important product information (e.g., Sanbonmatsu et al., 1994). With these results, we add to the current omission detection literature by showing that positive affect has a similar effect on omission detection as category expertise. Our results suggest that people in a positive affective state may be more likely to think about the details of the information presented to them and their existing scripts around what product information they would like to have and then form a desire for additional product information, which alters their evaluation of the product.

Of note, the products chosen for the current study represent those for which people are likely to have some existing product knowledge. Unlike the camera stimulus (Sanbonmatsu et al., 1992), which requires a high level of expertise to understand all product features, our chosen stimuli (e.g., pen, calculator, vacation, and restaurant) were products about which most people are familiar. Thus, product knowledge is a boundary condition that could be tested in future research. Perhaps positive affect would have no effect on omission detection when a product requires a high level of product knowledge that most people do not have (are less familiar) because they do not have a readily available internal schema about the product category. In this situation, they would not be able to rely on existing memories to identify gaps in the presented information.

### Limitations and future research directions

This research has some limitations, which should be considered. First, we focus on the processing characteristics of manipulated, incidental positive affect versus neutral affect and omit the study of naturally occurring affect (which is integral to the task) and negative affect. While our focus provides some insights into the influence of incidental positive affect on decision making, to investigate the processing characteristics of task induced affect and variations of positive and negative affect (happiness, pride, angry, sadness, etc.) more fully, a complex investigation into the different types of positive and negative emotion would be necessary, including an examination of the activation levels or motivation intensity of these affective states. For this reason, we leave the study of negative affect and task induced affect to future research.

Second, our affect manipulation looks at incidental positive affect versus neutral affect manipulated prior to the data collection task. We use a holdout sample to test the manipulation (experiments 1–3) to minimize the potential demand effect, however, in the fourth experiment, we overtly measure the manipulation in the main experiment in order to measure motivational intensity (or arousal) and ensure there is no significant differences across condition. The 9 × 9 grid (rather than three bi-polar scales) was used in order minimize the impact of the manipulation check. We did not measure mood using both a pre-task and a post-task manipulation check to ascertain if the mood induction was active during the entire data collection. Our task was relatively short, so we do not believe that the mood may have dissipated, but this could be studied in future research.

Third, our research focuses on a brand consumption situation because brand communications are relevant to all consumers and typically focused on only favorable (biased) presentation of brand related information. Thus, understanding how consumers process this incomplete information is good for public policy and consumer protection. However, it would be interesting to expand this research into other situations where partial or biased information is presented. For example, future research could look at social media memes, political communication, and other venues where it would be advantageous to correctly determine the completeness of the information.

## Conclusion and application

All in all, our results show that, positive affect increases (compared to neutral affect) sensitivity to missing information. This is a key determinant of whether or not consumers are likely to form strong judgments based on weak evidence. These results are important not only to marketers, but also to anyone who is trying to convey a message. These results also contribute to the understanding the implications of positive affect on information processing and provide suggestions as how best to convey message related information in different situations.

## Data availability statement

The datasets presented in this study can be found in online repositories. The names of the repository/repositories and accession number(s) can be found below: https://osf.io/ja2tu/?view_only=338fd2c161764cf392c1a0bb7bb7f3a9.

## Ethics statement

The studies involving human participants were reviewed and approved by Institutional Review Board, Office of Research, Integrity at Ball State University and Human Research Protection Program (HRPP), Office of Research Compliance at Indiana University. The participants provided their written informed consent to participate in this study.

## Author contributions

SM, TM-S, FK, and AB contributed to the conceptualization, methodology, analysis, data curation, and writing. SM supervised all stages of the project. All authors have read and agreed to the published version of the manuscript.
