# Supplementary material for: The influence of positive affect on sensitivity to important omissions
Source: Front Psychol. 2022 Nov 8;13:992489. doi: 10.3389/fpsyg.2022.992489 (PMC9680845; doi:10.3389/fpsyg.2022.992489)
Supplement: Supplementary file 1 [file Data_Sheet_1.pdf]

Supplementary Material

Appendix A

Experiment 2 design <u>within each cuing</u> condition.

	Session 1	Session 2
Group A	Pen, positive affect	Calculator, neutral affect
Group B	Pen, neutral affect	Calculator, positive affect
Group C	Calculator, positive affect	Pen, neutral affect
Group D	Calculator, neutral affect	Pen, positive affect

Within each cuing, the following 2x2x2 table can be constructed where subscripts are relevant to the analyses described below:

	Time 1		Time 2	
	Positive Affect	Neutral Affect	Positive Affect	Neutral Affect
Pen	A_1	B_1	D_2	C_2
Calculator	C_1	D_1	B_2	A_2

To analyze the data, two between-subjects variables were evaluated, one of which contrasted groups A and C with groups B and D (affect), and the other of which contrasted groups A and B with groups C and D (product). Specifically:

Dummy 1 (product): A_1, B_1 and C_2,D_2 (1) vs. A_2,B_2 and C_1, D_1 (-1)

Dummy 2 (affect): A_1, C_1 and B_2,D_2 (1) vs. A_2,C_2 and B_1, D_1 (-1)

Then, data were analyzed as a function of Dummy 1, Dummy 2, and Cuing Condition across Time using a repeated-measures ANOVA (SPSS GLM). Within each cuing condition, the main and interaction effects of conceptual interest are equivalent to the following statistical contrasts:

Main and interaction effect	Contrast
Time:	*Time*
Product:	*Dummy 1 * Time*
Affect:	*Dummy 2 * Time*
Product * Time:	*Dummy 1*
Affect * Time:	*Dummy 2*
Affect * Product* Time:	*Dummy 1 * Dummy 2 * time*

There were no main or interaction effects for Dummy 1 (Product), therefore all subsequent analyses are pooled across product -- that is, Groups A&C, and Groups B&D are combined.

Appendix B

Supporting analyses for Experiment 2:

It is important to think about the mediation analysis relative to the order of data collection. In our case, the proposed mediator was collected after the proposed dependent variable (purchase intent). Although, both process measures (description of the decision process and need for additional information) asked the participant to think back to the decision itself in answering the question. To evaluate the potential temporal order implications, the mediation analysis was run twice with each variable occupying the DV position and Mediator (M) in the various runs. The hypothesized model is also the model with the best fit (the proposed mediator as M and purchase intent as the DV) and is the model reported. Data available on the following link: https://osf.io/ja2tu/?view_only=338fd2c161764cf392c1a0bb7bb7f3a9 .

Follow-up correlations among the variables change across conditions such that in the neutral non-cued condition only the purchase intent and presented attribute rating were correlated (r= .734, p<.001), in the positive affect non-cued condition, purchase intent was correlated with presented attribute ratings (r= .503, p=.004) and need for additional information (r= .361, p=.046), and in the cued conditions purchase intent was correlated with presented attribute ratings (r= .433, p<.001) and omitted attribute ratings (i.e., the cue, r= .271, p=.03).

Appendix C

Experiment 4 stimuli

Cognitive product description:

Full information description

Krystal Bistro

Good tasting food
Quality food
Good service
Pleasant atmosphere
Moderately priced
Convenient location
Food selection that you cannot make at home
Not too crowded
Quiet atmosphere
Pleasant decor
Well decorated
Accepts reservations
Wine by the glass

Missing information description

Krystal Bistro

Good service
Pleasant atmosphere
Not too crowded
Quiet atmosphere
Pleasant decor
